# Three-dimensional printing models increase inter-rater agreement for classification and treatment of proximal humerus fractures

**DOI:** 10.1186/s13037-021-00312-7

**Published:** 2022-01-20

**Authors:** Luiz Fernando Cocco, André Yui Aihara, Flávia Paiva Proença Lobo Lopes, Heron Werner, Carlos Eduardo Franciozi, Fernando Baldy dos Reis, Marcus Vinicius Malheiros Luzo

**Affiliations:** 1 Department of Orthopedic, Escola Paulista de Medicina, Universidade Federal de São Paulo, Hospital Samaritano Higienópolis Américas Serviços Médicos, São Paulo, Brasil; 2 Department of Diagnostic Imaging, Escola Paulista de Medicina, Universidade Federal de São Paulo, São Paulo, Brasil; 3 Diagnósticos da América, São Paulo, Brasil; 4 Department of Orthopedic, Orthopaedic Surgeon, Escola Paulista de Medicina, Universidade Federal de São Paulo, São Paulo, Brasil

**Keywords:** Proximal humerus fractures, 3D printing models, Shoulder fractures, Diagnostic accuracy, Inter-rater agreement

## Abstract

**Background:**

Proximal humerus fractures (PHF) are frequent, however, several studies show low inter-rater agreement in the diagnosis and treatment of these injuries. Differences are usually related to the experience of the evaluators and/or the diagnostic methods used. This study was designed to investigate the hypothesis that shoulder surgeons and diagnostic imaging specialists using 3D printing models and shoulder CT scans in assessing proximal humerus fractures.

**Methods:**

We obtained 75 tomographic exams of PHF to print three-dimensional models. After, two shoulder surgeons and two specialists in musculoskeletal imaging diagnostics analyzed CT scans and 3D models according to the Neer and AO/OTA group classification and suggested a treatment recommendation for each fracture based on the two diagnostic methods.

**Results:**

The classification agreement for PHF using 3D printing models among the 4 specialists was moderate (global k = 0.470 and 0.544, respectively for AO/OTA and Neer classification) and higher than the CT classification agreement (global k = 0.436 and 0.464, respectively for AO/OTA and Neer). The inter-rater agreement between the *two shoulder surgeons* were substantial. For the AO/OTA classification, the inter-rater agreement using 3D printing models was higher (k = 0.700) than observed for CT (k = 0.631). For Neer classification,  inter-rater agreement with 3D models was similarly higher (k = 0.784) than CT images (k = 0.620). On the other hand, the inter-rater agreement between the *two specialists* in diagnostic imaging was moderate. In the AO/OTA classification, the agreement using CT was higher (k = 0.532) than using 3D printing models (k = 0.443), while for Neer classification, the agreement was similar for both 3D models (k = 0.478) and CT images (k = 0.421). Finally, the inter-rater agreement in the treatment of PHF by the 2 surgeons was higher for both classifications using 3D printing models (AO/OTA—k = 0.818 for 3D models and k = 0.537 for CT images). For Neer classification, we saw k = 0.727 for 3D printing models and k = 0.651 for CT images.

**Conclusion:**

The insights from this diagnostic pilot study imply that for shoulder surgeons, 3D printing models improved the diagnostic agreement, especially the treatment indication for PHF compared to CT for both AO/OTA and Neer classifications On the other hand, for specialists in diagnostic imaging, the use of 3D printing models was similar to CT scans for diagnostic agreement using both classifications.

**Trial registration:**

Brazil Platform under no. CAAE 12273519.7.0000.5505.

## Introduction

Proximal humerus fractures (PHF) are frequent, affecting a significant number of adults and elderly victims due to trauma or falls. Its prevalence in hospital emergency care is substantial and corresponds to approximately 45% of humerus fractures and 5% of total fractures [12, 23, 29]. However, understanding these fractures and the best way to treat them remains unsettled between doctors and researchers [8, 9, 11, 22, 24, 25]..

Although PHF is relevant and is growing worldwide, controversies related to its diagnosis and treatment definitions are still frequent [8, 9, 11, 17, 22, 25]. The classifications proposed by Charles Neer [22] and the AO/OTA group—Arbeit Gemeinschaft für Osteosynthesefragen [17] are widespread and used worldwide. However, there is still no relevant reproducibility for the diagnosis and treatment of PHF [1, 3, 5–7, 10, 15, 21]..

This study presents 3D printing models of PHF as an alternative method for diagnosing and treating these injuries. These models, also called prototypes, are personalized and individualized prints reproducing three-dimensionally and faithfully the fractures' original characteristics. We believe that the 3D models created will help understanding the patterns of shoulder fractures, improving their classification and, consequently, the treatment.

Therefore, this work analyzes the inter-rater agreement and the interface between shoulder surgeons and specialists in musculoskeletal imaging, comparing computed tomography exams with 3D printing models.

## Methods

We obtained 75 tomographies of fractures of the proximal humerus at random from an image database of Hospital Samaritano Higienópolis -Américas Serviços Médicos. There was no identification of the patients submitted to the exams, and confidentiality and anonymity were maintained throughout the study. We included tomographic images of fractures of the proximal humerus of both sexes, adults (physeal growth plate closure checked), attended at the Hospital with complete and good quality tomographic exams (sagittal, axial, and coronal sections). We did not include images related to pathological (neoplastic) fractures, infectious diseases (acute or chronic), pre-existing PHF or deformities, and congenital morphological changes.

**Fig. 1:**
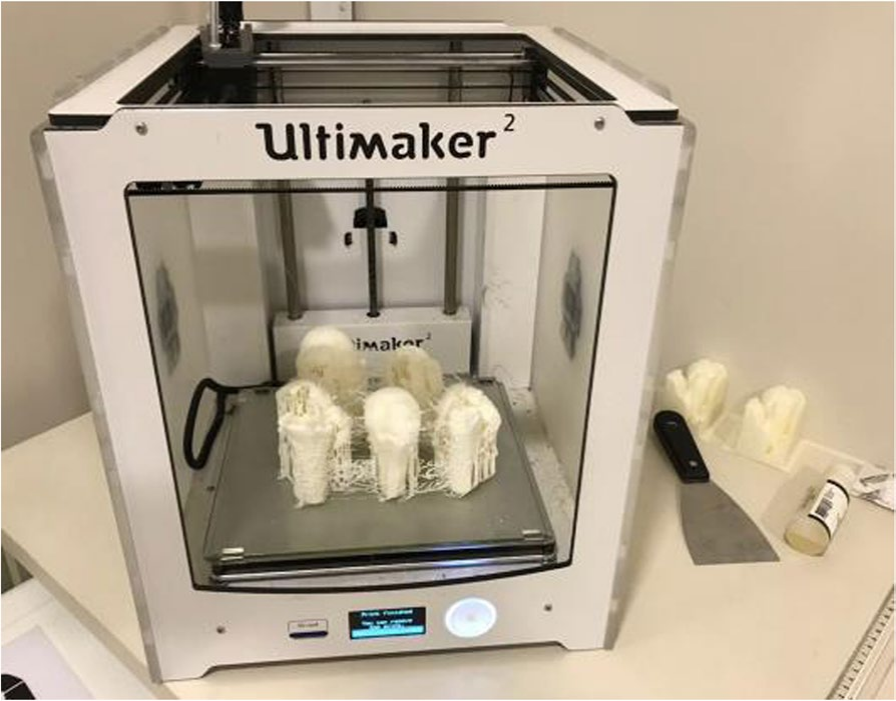
3D printing models of proximal humerus fractures using CT scans as a model

**Fig. 2:**
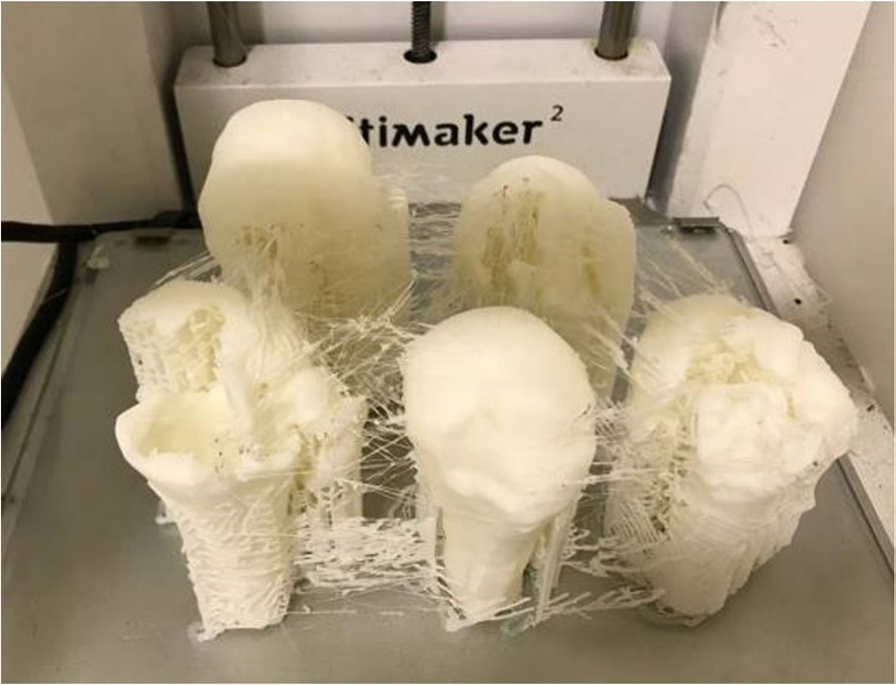
3D printing models of proximal humerus fractures using CT scans as a model

**Fig. 3:**
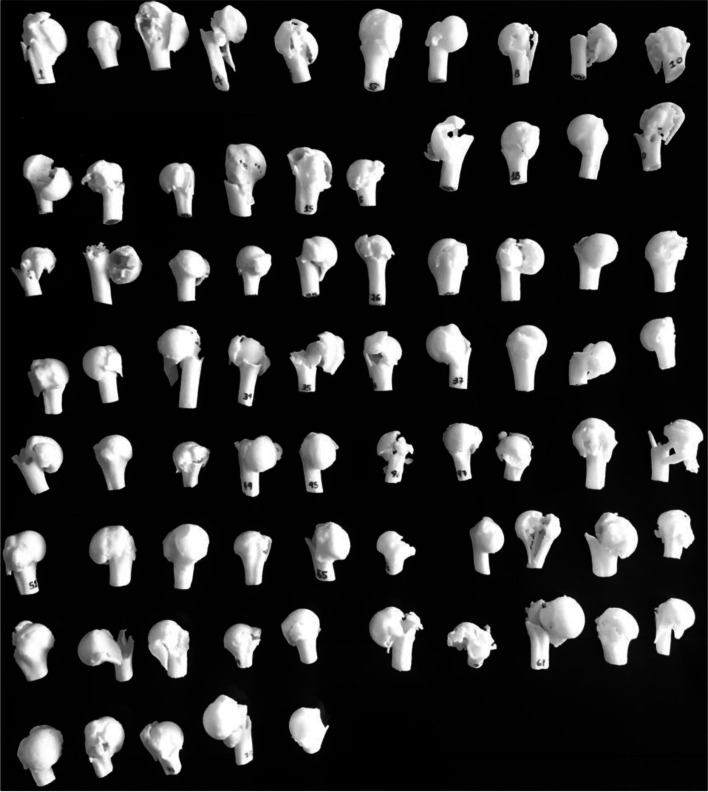
Photograph of 75 samples of 3D printing models of proximal humerus fractures

The 75 selected tomographic images were used for three-dimensional printing models (prototypes) of the fractures, with a single piece corresponding to each image. The company DASA (Diagnósticos da América) executed the prints and donated them to the researchers. The pieces were printed in PLA (polyacid lactic), a synthetic thermoplastic polymer of biological origin, obtained by renewable resources, composed of starch or sugar such as corn, wheat, beet, or sugar cane. The models were printed in actual size, reproducing exclusively the bone characteristics of the fractures, with no inclusion of the scapula or clavicle (Figs. 1, 2 and 3). Complex fractures were not excluded to maintain the original characteristics of each case, however we do not include the scapula and clavicle in the three-dimensional models. The impressions would result in a unique model, making the scapulohumeral joint limits imprecise for three-dimensional analysis. Furthermore, the coverage of part of the humeral head by fusion in the glenoid would not allow the assessment of joint fractures, interfering in the classification and especially in the therapeutic indications. For this reason, we did not include images of fractures dislocations of the shoulder, so that parameters of joint inconsistencies were not considered by the experts.

**Fig. 4:**
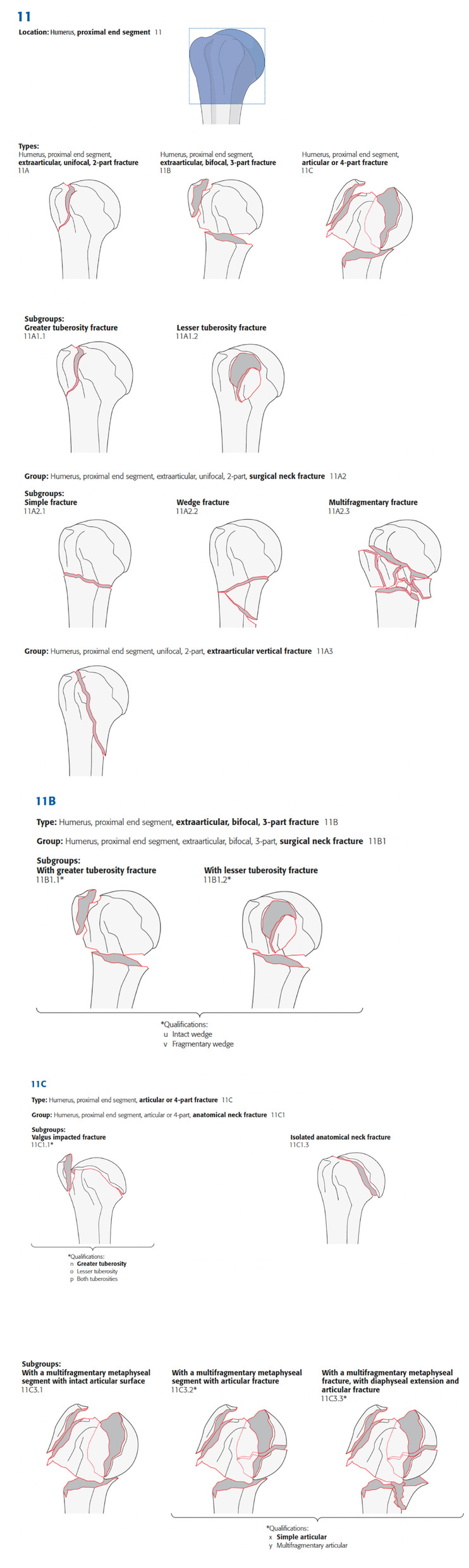
AO/OTA classification for fractures of the proximal humerus. Obtained from Meinberg EG et al. (pS1-S10) [16]

**Fig. 5:**
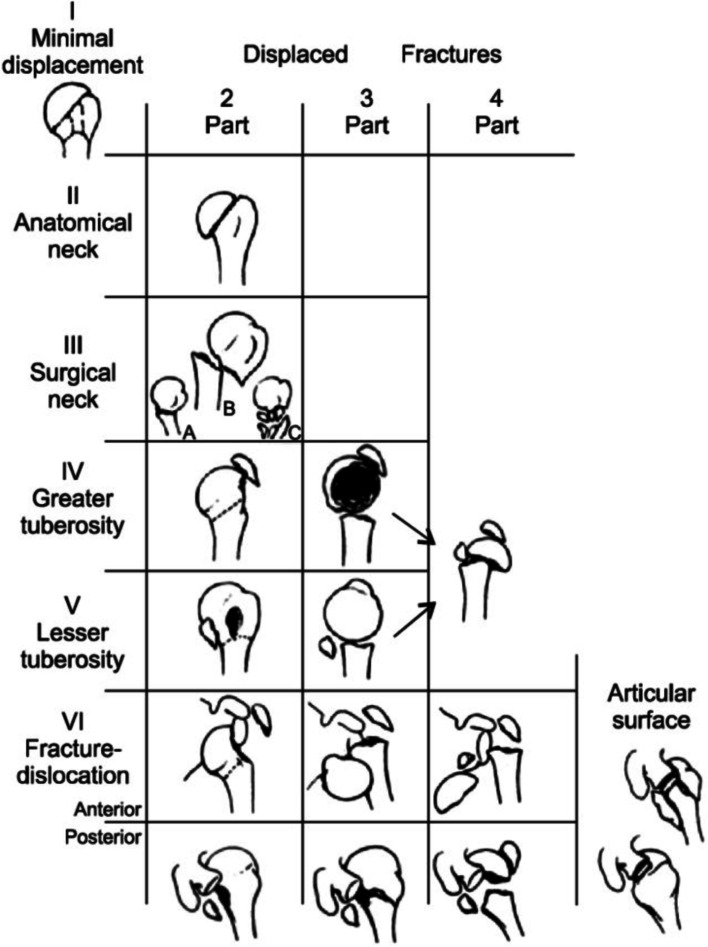
Neer’s classification of proximal humeral fractures. Neer CS 2^nd^. Displaced proximal humeral fractures. Part I. Classification and evaluation. J Bone Joint Surg Am. 1970;52(6):1077–89 [21]

Two orthopedists, specialists in shoulder surgery linked to the Shoulder and Elbow Sector of the Department of Orthopedics and Traumatology at Escola Paulista de Medicina (DOT/UNIFESP) and two doctors, specialized in musculoskeletal imaging diagnostics associated with the Department of Diagnostic Imaging at Escola Paulista de Medicina (DDI/UNIFESP), were invited to evaluate the exams. The four doctors had at least 5 years of experience in their respective areas. They did not participate in the selection of tomographic images from the database or in the 3D model printing. Before the beginning of the evaluations, the four experts participated in a theoretical review on the concepts of the adopted classifications and received the AO/OTA and Neer classifications sheets to be used or consulted during the procedure (Figs. 4 and 5).

**Fig. 6:**
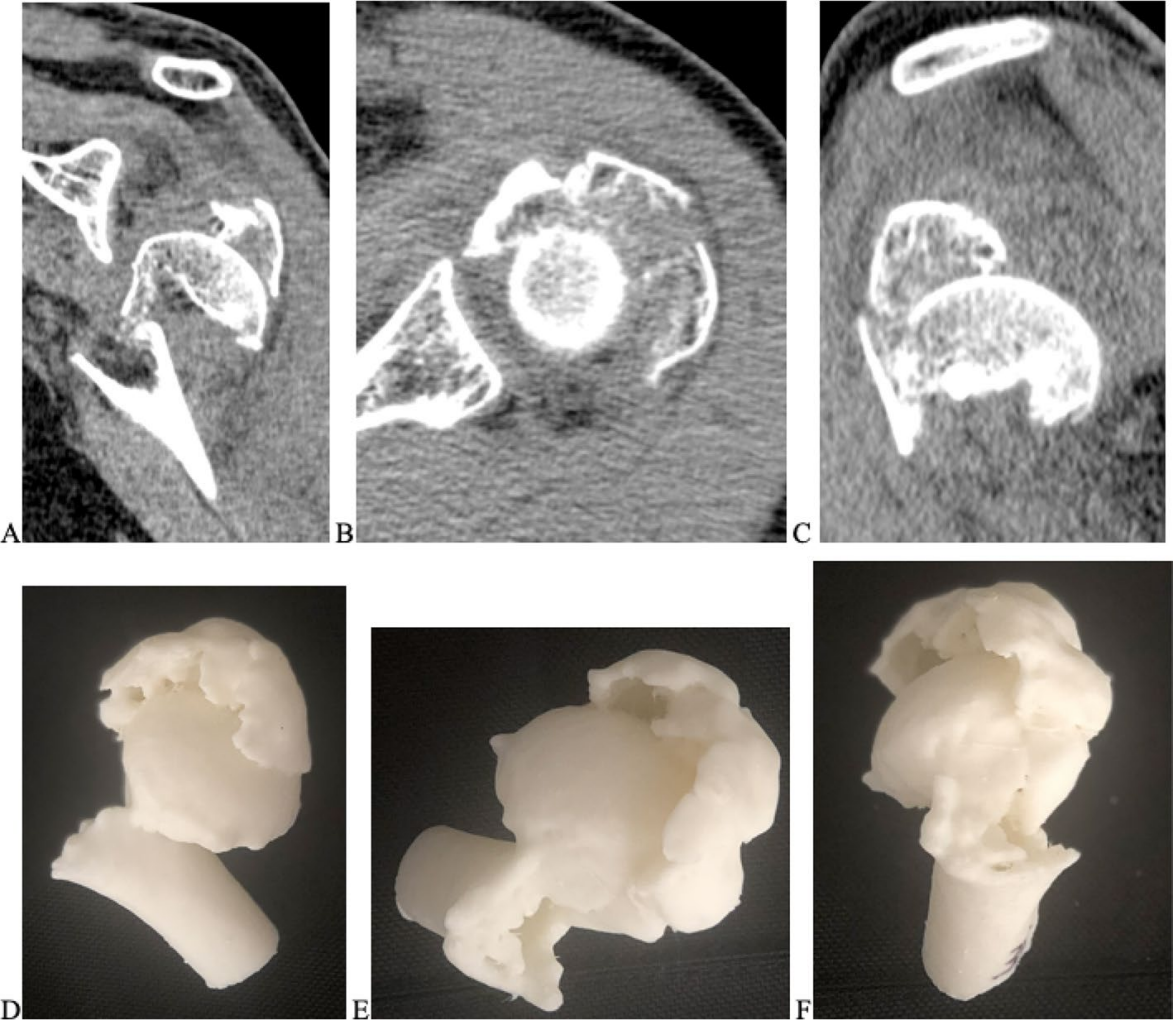
Case 35 of PHF with tomographic images and frontal (**A**), axial (**B**), and sagittal (**C**) sections. 3D models in frontal view (**D**), axial (**E**), and sagittal (**F**)

The tomographic images and the 3D models were evaluated simultaneously by the four doctors at different times. The 75 tomographies were displayed sequentially on high-resolution screens, as axial, coronal, and sagittal complete sections, so that doctors could classify the fractures according to the AO/OTA classification. Later the specialists received each of the three-dimensional models corresponding to the tomographic images in sequential order. The prototypes were randomly delivered in relation to the previously presented tomography cases, so they could also be classified according to the AO/OTA classification. Then, the same protocol was followed, adopting the Neer classification. The Fig. 6 (case 35) exemplifies some correlations between 3D printing models and corresponding tomographic images to be compared.

The evaluations were carried out using multiple-choice questionnaires and individualized by classification (Tables 1 and2 ).

The four specialists were responsible for classifying the fractures according to AO/OTA and Neer classification using the two diagnostic methods. However, only shoulder surgeons were asked to indicate any treatment for each case. The options were divided into two indications: non-surgical and surgical (osteosynthesis or shoulder arthroplasty). During the evaluation of the images, no information regarding clinical history, sex, age, upper limbs dominance, or the patients' possible associated diseases was disclosed. To maintain a correspondence between the AO/OTA and Neer classifications in relation to the number of parts (type A—AO/OTA equivalent to 2-parts of Neer, and types B and C—AO/OTA equivalent to 3- and 4-parts of Neer, respectively) as proposed by Meinberg et al [17], we excluded the AO/OTA classification subtypes in this study, maintaining the correspondence confidence that concordance studies require.

After two weeks, the same four specialists were invited to repeat the assessments, using similar protocol and conditions mentioned above for the same 75 cases, for results’ reproducibility.

### Statistical analysis

We used a 95% confidence interval for the analysis with a sampling error of 0.07 for a Kappa concordance coefficient estimated at 0.50**.**For this calculation, a standard deviation of 0.30 was used [13]. For these calculations, we used the statistical software PASS 2008 (Power Analysis and Sample Size System)—NCSS.

The evaluation of inter-observer and intra-observer agreement was performed using Kappa coefficients. The overall Kappa coefficients were shown to classify the agreement between more than two observers. For all statistical tests, a significance level of 5% was used.

Statistical analyzes were performed using the statistical software SPSS 20.0 and STATA 12.

## Results

Kappa coefficients between both classifications (AO/OTA and Neer), treatment indication, and diagnostic method are shown (Table 3). The inter-rater agreement for PHF using 3D printing models among the *four specialists* was moderate (overall k = 0.470 and 0.544, respectively for AO/ OTA and Neer classification), however, higher than CT (overall k = 0.436 and 0.464, respectively, for AO / OTA and Neer classification).

The classifications between the *two shoulder surgeons* were substantial. In the AO/OTA classification, the inter-rater agreement using 3D printing models was higher (k = 0.700) than seen for CT (k = 0.631). For Neer classification, inter-rater agreement with 3D models was higher (k = 0.784) than CT images (k = 0.620). Moreover, the inter-rater agreement between the *two specialists* in diagnostic imaging was moderate. In the AO/OTA classification, the  inter-rater agreement using CT was higher (k = 0.532) than using 3D printing models (k = 0.443). Using the Neer classification, the inter-rater agreement was similar for both 3D models (k = 0.478) and CT images (k = 0.421).

The two surgeons' inter-rater agreement for treatment indication of PHF was higher for both classifications using 3D printing models. In the AO/OTA classification, the inter-rater agreement for 3D models was k = 0.818 (almost perfect), while for CT was k = 0.537 (moderate). The Neer inter-rater agreement for treatment indication with 3D models was k = 0.727 and k = 0.651 for CT images (both were substantial).

### Concordance between classification types (AO/OTA and Neer) by specialist and diagnostic method

In this analysis, the 68 images that did not obtain the classification as 1-part fracture in the Neer Classification were considered. In this way, it was possible to maintain the fracture’s correspondence between both classifications due to the absence of correspondence between the 1-part fractures for Neer and AO/OTA classifications [17].

As seen in Table 4, surgeons had a higher inter-rater agreement between AO/OTA and Neer classifications using 3D printing models than CT images. For the specialists in diagnostic imaging, the inter-rater agreement was similar for both 3D and CT.

### Reproducibility between different periods of evaluation after a 15-day interval

There were substantial to almost perfect reproducibilities using CT images and 3D models (Kappa values ranged from 0.615 to 0.839, respectively) for both images’ classification and treatment indication (Table 5). Among specialists in diagnostic imaging, a moderate inter-rater agreement was seen for fractures classification using 3D printing models (Kappa values ranged from 0.410 to 0.459). On the other hand, for CT images, one specialist showed moderate and the other weak concordance.

## Discussion

In this work, among surgeons, the association of 3D printing models and the AO/OTA and Neer classifications improved the intra- and inter-observer agreement for the diagnosis of PHF compared with CT scans. Moreover, as seen in a previous publication [1, 3, 5], treatment indication had the best inter-rater agreement between surgeons. On the other hand, among specialists in musculoskeletal imaging, 3D printing models and CT scans showed moderate inter-rater agreement between the diagnostic methods. The descriptive pattern of fractures for specialists in musculoskeletal imaging daily routine usually focuses on local anatomical characterization [16]. Moreover, they are unfamiliar with orthopedic classifications used by surgeons (AO/OTA and Neer), explaining the divergent results. We believe that treatment planning using 3D printing models facilitates surgeons' diagnosis when manipulating the bone fragments and implants' choice (size and models of plates or nails, and the number of screws). Besides, the surgeon can objectively understand bone imperfections that are frequent in fractures of the humerus. Surgeons are influenced by tactile rather than exclusively visual aspects of shoulder fractures. The manipulation of 3D models ends up stimulating reasoning and interpretation areas that may not be required by visual exams only, such as CT scans. Similar to 3D model manipulation, palpation of bone fragments are part of the surgical procedure for fracture pattern understanding. In this respect, only 3D printing models can reproduce this stimulus, explaining the higher inter-rater agreement obtained for treatment indication reported here.

Shoulder surgeons still diagnose and choose treatments based on their own experience and training, with weak evidence in most cases. Slings, plates, nails, and prostheses are present in their therapeutic arsenal to correlate the patient's characteristics, the fracture, and the surgeon's ability to use each one. On the other hand, specialists in imaging diagnosis have similar difficulties in diagnosing these fractures, either due to high lesion variability or the descriptive training in image interpreting. Also, they are unfamiliar with pre-existing classifications such as AO/OTA or Neer classification, unlike shoulder surgeons, leading to opinion and diagnostic divergences.

Although there is no gold standard for diagnosing PHF, radiographs and CT scans are widely used for initial evaluation. Radiographs are cheap and quick and may show patients' critical characteristics related to shoulder pain and after local trauma. However, positioning the patient with pain during examination impairs the diagnosis and the correct interpretation of the fracture. Thus, in clinical practice, tomography is widely used to assess and characterize the extent of shoulder fractures [2, 4, 18]. Besides, it is through these exams that information for treatment choice is observed.

In parallel with scientific development in the medical area, 3D models in the industrial and daily lives are frequent. 3D printers can turn palpable images previously imprisoned on screens increasingly popular in quality and costs. This area's evolution is also growing in the clinical and medical field scientific routine[27]. Research on the particularities of several fractures involving three-dimensional prototypes is growing, and the results stimulate more applications. In the orthopedic area, 3D models of fractures can improve the understanding of injuries' complexity among specialists and assist in educating doctors and professionals involved in the treatment of these diseases [14, 19, 20, 26–28, 30].

Among specialists in musculoskeletal imaging, 3D printing models and CT scans showed moderate inter-rater agreement between the diagnostic methods. Thus, grouping fractures within the proposed classifications may have been harder for them compared to surgeons. According to Mitsouras et al [19], the inter-rater agreement between different methods, the inclusion of 3D printing models in the professional routine of orthopedic classifications, and the possibility of accessing these models will bring an essential alternative for evaluating humerus fractures by specialists in diagnostic imaging.

Although 3D printing models are not yet considered an official diagnostic method, and may add costs and time (around US$ 30,00/each and 90 min for each printing) to the diagnostic process, they reproduce reliable prototypes from CT images. For the medical field, anatomical parts' characterization and the customization and optimization of resources can improve orthopedical diseases' diagnosis and treatment. In addition, the 3D models can be used for training and for educating doctors and health professionals. Our work shows these interfaces, improving the understanding of PHF treatment among specialists and surgeons.

However, we emphasize that although this study was designed to analyze the inter-rater agreement between 3D impression models, CT scans, classifications and experts, the lack of information related to sensitivity and specificity between the methods does not allow us to discuss a possible superiority between the exams. In addition, other limitations such as absence of patients clinical information, or excluding some patterns of shoulder fractures (fractures dislocations) may have influenced surgeons in the choice of treatment for each case presented.

## Conclusions

For shoulder surgeons, 3D printing models improved the diagnostic, especially the inter-rater treatment indication agreement for PHF compared to CT scans for both AO/OTA and Neer classifications.

For specialists in diagnostic imaging, the use of 3D printing models was similar for diagnostic inter-rater agreement of PHF compared to CT for both AO/OTA and Neer classifications.

**Table 1:** Questionnaires for shoulder surgeons

Tomography		
Cases	AO/OTA Classification	Treatment A: Non-surgical B: Osteosynthesis C: Arthroplasty
1	A B C	A B C
2	A B C	A B C
3	A B C	A B C
4	A B C	A B C
5	A B C	A B C
Up to 75	A B C	A B C
3D printing models		
Cases	AO/OTA Classification	Treatment A: Non-surgical B: Osteosynthesis C: Arthroplasty
1	A B C	A B C
2	A B C	A B C
3	A B C	A B C
4	A B C	A B C
5	A B C	A B C
Up to 75	A B C	A B C
Tomography		
Cases	Neer classification (number of parts)	Treatment A: Non-surgical B: Osteosynthesis C: Arthroplasty
1	1 2 3 4	A B C
2	1 2 3 4	A B C
3	1 2 3 4	A B C
4	1 2 3 4	A B C
5	1 2 3 4	A B C
Up to 75	1 2 3 4	A B C
3D printing models		
Cases	Neer classification (number of parts)	Treatment A: Non-surgical B: Osteosynthesis C: Arthroplasty
1	1 2 3 4	A B C
2	1 2 3 4	A B C
3	1 2 3 4	A B C
4	1 2 3 4	A B C
5	1 2 3 4	A B C
Up to 75	1 2 3 4	A B C

**Table 2:** Questionnaires for diagnostic imaging specialists

Tomography	
Cases	AO/OTA Classification
1	A B C
2	A B C
3	A B C
4	A B C
5	A B C
Up to 75	A B C
3D printing models	
Cases	AO/OTA Classification
1	A B C
2	A B C
3	A B C
4	A B C
5	A B C
Up to 75	A B C
Tomography	
Cases	Neer classification (number of parts)
1	1 2 3 4
2	1 2 3 4
3	1 2 3 4
4	1 2 3 4
5	1 2 3 4
Up to 75	1 2 3 4
3D printing models	
Cases	Neer classification (number of parts)
1	1 2 3 4
2	1 2 3 4
3	1 2 3 4
4	1 2 3 4
5	1 2 3 4
Up to 75	1 2 3 4

**Table 3:** Kappa coefficient by type of classification, treatment indicated, and methods of diagnosis

	AO/OTA					Neer			
	3D Models		CT			3D Models		CT	
	Kappa	p	Kappa	p		Kappa	p	Kappa	p
Classification					Classification				
All specialists	0.470	< 0.001	0.436	< 0.001	All specialists	0.544	< 0.001	0.464	< 0.001
Fractures Type A	0.653	< 0.001	0.642	< 0.001	1-part	0.602	< 0.001	0.733	< 0.001
Fractures Type B	0.363	< 0.001	0.241	< 0.001	2-parts	0.606	< 0.001	0.581	< 0.001
Fractures Type C	0.383	< 0.001	0.465	< 0.001	3-parts	0.465	< 0.001	0.326	< 0.001
					4-parts	0.538	< 0.001	0.444	< 0.001
Shoulder surgeons	0.700	< 0.001	0.631	< 0001	Shoulder surgeons	0.784	< 0.001	0.620	< 0.001
Diagnostic imaging specialists	0.443	< 0.001	0.532	< 0.001	Diagnostic imaging specialists	0.478	< 0.001	0.421	< 0.001
Treatment					Treatment				
Shoulder surgeons	0.818	< 0.001	0.537	< 0.001	Shoulder surgeons	0.727	< 0.001	0.651	< 0.001

*N* = 75 images

**Table 4:** Kappa coefficient of agreement between classifications and diagnostic methods among specialists for proximal humerus fractures

AO/OTA versus Neer	3D Models		CT	
	Kappa	p	Kappa	p
Shoulder surgeon 1	0.518	< 0.001	0.442	< 0.001
Shoulder surgeon 2	0.341	< 0.001	0.260	0.001
Specialist in diagnostic imaging 1	0.315	< 0.001	0.327	< 0.001
Specialist in diagnostic imaging 2	0.443	< 0.001	0.477	< 0.001

**Table 5:** Reproducibility of Kappa coefficients between different periods of evaluation by type of classification, diagnostic methods, and between specialists. Interval of 15 days

	3D Models		CT	
	Kappa	p	Kappa	p
Surgeon 1				
AO / OTA Classification	0.839	< 0.001	0.735	< 0.001
Neer Classification	0.747	< 0.001	0.650	< 0.001
AO/OTA Treatment	0.742	< 0.001	0.762	< 0.001
Neer Treatment	0.618	< 0.001	0.615	< 0.001
Surgeon 2		< 0.001		
AO / OTA Classification	0.750	< 0.001	0.710	< 0.001
Neer Classification	0.727	< 0.001	0.742	< 0.001
AO/OTA Treatment	0.767	< 0.001	0.818	< 0.001
Neer Treatment	0.756	< 0.001	0.720	< 0.001
Image Specialist 1		< 0.001		< 0.001
AO / OTA Classification	0.459	< 0.001	0.374	< 0.001
Neer Classification	0.410	< 0.001	0.386	< 0.001
Image Specialist 2		< 0.001		
AO / OTA Classification	0.429	< 0.001	0.578	< 0.001
Neer Classification	0.447	< 0.001	0.455	< 0.001

*N* = 75

## Data Availability

The datasets used and analysed during the current study are available from the corresponding. The authors declare that this research was carried out in strict compliance with all methods in accordance with the relevant guidelines and regulations proposed to ensure that ethical and moral principles have been carefully adopted and obeyed, and that meet the values ​​and ideals proposed by the editorial board of this journal.

## Abbreviations

PHF: Proximal humerus fracture
AO/OTA: Arbeit Gemeinschaft für Osteosynthesefragen group
DASA: Diagnósticos da América
PLA: Polyacid lactic
CT: Computed tomography
DOT/UNIFESP: Department of Orthopedics and Traumatology at Escola Paulista de Medicina
DDI/UNIFESP: Department of Diagnostic Imaging at Escola Paulista de Medicina
PASS: Power Analysis and Sample Size System
